# A Natural Language Processing (NLP) Evaluation on COVID-19 Rumour Dataset Using Deep Learning Techniques

**DOI:** 10.1155/2022/6561622

**Published:** 2022-09-14

**Authors:** Rubia Fatima, Naila Samad Shaikh, Adnan Riaz, Sadique Ahmad, Mohammed A. El-Affendi, Khaled A. Z. Alyamani, Muhammad Nabeel, Javed Ali Khan, Affan Yasin, Rana M. Amir Latif

**Affiliations:** ^1^School of Software, Tsinghua University, Beijing, China; ^2^Government Degree College for Women, Bosan Road, Multan, Pakistan; ^3^Department of Creative Technologies, Faculty of Computing and Artificial Intelligence, Air University, Islamabad, Pakistan; ^4^EIAS—Data Science and Blockchain Laboratory, College of Computer and Information Sciences, Prince Sultan University, Riyadh 11586, Saudi Arabia; ^5^Department of Computer Sciences, Bahria University Karachi Campus, Karachi, Pakistan; ^6^Applied College, Abqaiq Branch, King Faisal University, P.O. Box 4000, Al-Ahsa 31982, Hofuf, Saudi Arabia; ^7^School of Software Engineering, South China University of Technology, Guangzhou, China; ^8^Department of Software Engineering, University of Science and Technology Bannu, Bannu, Pakistan; ^9^Department of Computer Science, COMSATS University Islamabad, Sahiwal Campus, Islamabad, Pakistan

## Abstract

*Context and Background*: Since December 2019, the coronavirus (COVID-19) epidemic has sparked considerable alarm among the general community and significantly affected societal attitudes and perceptions. Apart from the disease itself, many people suffer from anxiety and depression due to the disease and the present threat of an outbreak. Due to the fast propagation of the virus and misleading/fake information, the issues of public discourse alter, resulting in significant confusion in certain places. Rumours are unproven facts or stories that propagate and promote sentiments of prejudice, hatred, and fear. *Objective*. The study's objective is to propose a novel solution to detect fake news using state-of-the-art machines and deep learning models. Furthermore, to analyse which models outperformed in detecting the fake news. *Method*. In the research study, we adapted a COVID-19 rumours dataset, which incorporates rumours from news websites and tweets, together with information about the rumours. It is important to analyse data utilizing Natural Language Processing (NLP) and Deep Learning (DL) approaches. Based on the accuracy, precision, recall, and the f1 score, we can assess the effectiveness of the ML and DL algorithms. *Results*. The data adopted from the source (mentioned in the paper) have collected 9200 comments from Google and 34,779 Twitter postings filtered for phrases connected with COVID-19-related fake news. *Experiment 1.* The dataset was assessed using the following three criteria: veracity, stance, and sentiment. In these terms, we have different labels, and we have applied the DL algorithms separately to each term. We have used different models in the experiment such as (i) LSTM and (ii) Temporal Convolution Networks (TCN). The TCN model has more performance on each measurement parameter in the evaluated results. So, we have used the TCN model for the practical implication for better findings. *Experiment 2.* In the second experiment, we have used different state-of-the-art deep learning models and algorithms such as (i) Simple RNN; (ii) LSTM + Word Embedding; (iii) Bidirectional + Word Embedding; (iv) LSTM + CNN-1D; and (v) BERT. Furthermore, we have evaluated the performance of these models on all three datasets, e.g., veracity, stance, and sentiment. Based on our second experimental evaluation, the BERT has a superior performance over the other models compared.

## 1. Introduction

In December 2019, the pandemic for 2019s novel Coronavirus (COVID-19) in Wuhan, China, became a worldwide severe public health problem [1, 2]. “An extreme acute respiratory syndrome called SARS-CoV-2 [3] has often been called the COVID-19 pandemic virus. Coronavirus (CoV) is a broad family of viruses that cause cold diseases, such as Middle East Respiratory Syndrome (MERS-CoV) and SARS-CoV.” The latest strain detected in 2019 and not commonly associated with human illness is COVID-19. Regardless of transmission from humans to wildlife, coronaviruses are zoonotic (a disease that can be transmitted to humans from animals). Studies suggest that a SARS-CoV infection from cats to humans is being transmitted and that the MERS-CoV is tainted with dromedary infection in humans [4]. The virus COVID-19 is believed to be bats-to-human infection. The widespread distribution of the virus culminated in the pulmonary transfer of the disease from person to person. Although, in approximately 82% of cases COVID-19 induces milder effects, some are severe or critical [5]; dyspnoea (shortness of breath), cough, and fever are indicators of infection.

Genome sequencing of respiratory or blood samples may be used to confirm the COVID-19 diagnosis as a major predictor for RT-PCR or in-patient treatment. Although RT-PCR shows poor resistance, many COVID-19 patients remain undetected and unmanageable. Furthermore, the danger of infecting a larger population due to the virus' high contagiousness cannot be undermined [6]. It is therefore critical that artificial intelligence capabilities be leveraged through the use of ultrasound, X-rays, and computed tomography images through emerging medical care systems that improve diagnostics of COVID-19 [7–9].

Today, diagnosis covers anyone who displays the famous pneumonia trend in COVID-19 chest scan, rather than search for successful results. Our proposed approach would enable policymakers to separate patients and manage them faster. Many people recover with constant lung injury even though there is no mortality with COVID-19. COVID-19 also has the lungs like SARS, which gives them a “honeycomb-like look,” according to the World Health Organization. On the one side, artificial learning leads to product development and, on the other side, to handle global crises. The COVID-19 treatment includes medical instruments and professional personnel who are subject to elevated risks themselves because there is no appropriate managed environment.

Scholars have focused on machine learning NLP methods to prevent the propagation of disinformation [10]. Soni and Roberts [11] identified BERT model with very little preprocessing text, yet achieved excellent efficiency. Facebook deleted more than 50 million posts linked to COVID-19 by April 2020 since they were identified as disinformation using NLP-based machine learning methods. Using these deep learning algorithms [12, 13], social media companies like Twitter and Google have also taken down adverts, and fraudulent posts related to COVID-19 [14].

Although attempts have been carried out utilizing deep learning models to identify COVID-19 disinformation, there has been a scarcity of research on how ordinary people might simultaneously recognize false information and boost their confidence [15]. Furthermore, black-box models are frequently used in machine-learning-based NLP approaches. Explainable AI in high-risk decision-making is more important in other areas of medicine, such as COVID-19 and fatigue detection. On the other hand, if these models offer insights, they may assist in increasing confidence and acceptance as well as achieving the desired goals [14].

This study utilized several deep learning algorithms, such as LSTM networks, which are redundant neural networks that can learn order dependence in sequence prediction issues. It is necessary for complicated problem areas like machine translation and voice recognition to utilize DL approaches [16, 17].

The key papers initially suggested the video segmentation of the Temporal Convolutional Networks (TCNs). The two stages in this traditional method involve low-level calculating functions using CNN [18–20] to encode spatial-temporal information and introduce low-level functions into a classifier, which captures time information at high levels using (generally) RNN. A similar method needs two distinct models, which is the major drawback [21]. TCN offers a unified method to capture all two information layers (encoder-decoder) hierarchically.

The spread of false information concerning COVID-19 poses a severe risk to public health [22]. Roozenbeek et al. [23] investigate common misconceptions regarding the virus and look into the factors that influence people's willingness to accept the most widely spread falsehoods. The authors also find that people's compliance with public health guidelines concerning COVID-19 is negatively affected by their susceptibility to misinformation.

**Figure 1:**
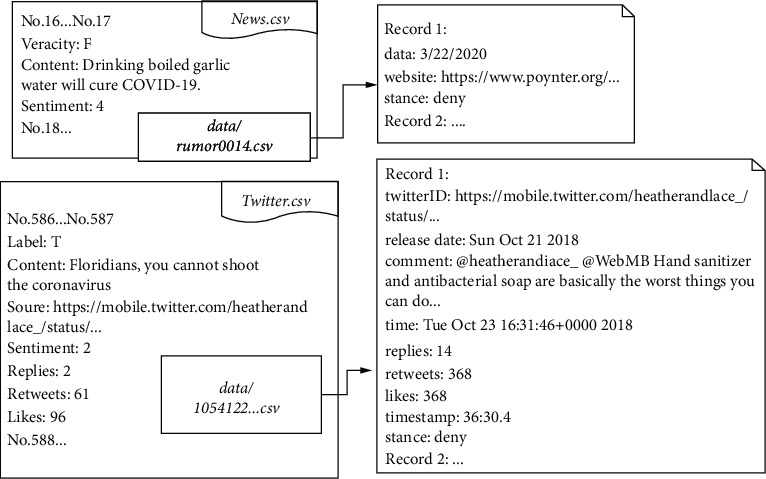
Examples of data structure adopted from [24].

COVID-19 is thus still in significant need of rumours to analyse mood and other rumour categorization activities, including position verification of COVID-19 rumours. We gathered COVID-19 9000+ news rumours and 34,000+ tweets with feelings and labels of position for the research study.  Figure1illustrates examples of our adapted statistics and data structure. We also analysed our dataset using statistical analysis of rumour propagation and classification findings for a deeper learning rumour classification.

**Figure 2:**
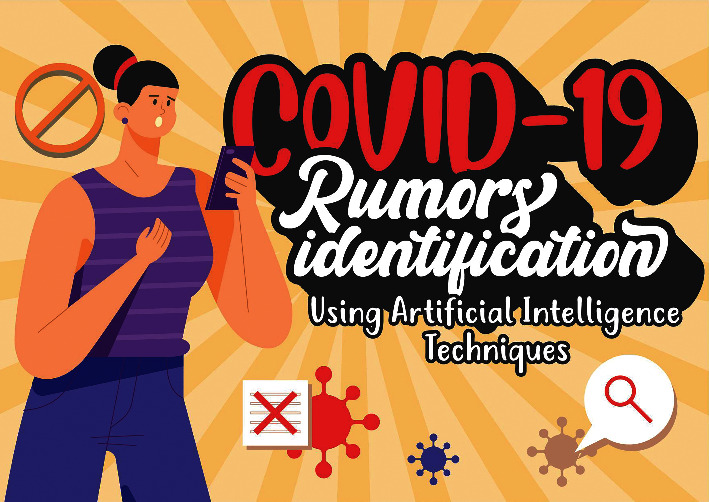
COVID-19 rumour identification using AI techniques.

This paper (Figure 2) is divided into five sections. The first part is an introduction to the work that we want to do. The literature review of pertinent research is presented in the second part. The technique and data gathering process are discussed in the third part. The results and discussion of the entire study are presented in the fourth part. We have come to the end of the research in the fifth segment.

## 2. Literature Review

As a result of this study's usage of COVID-19 from Sina Weibo, it is possible to identify rumours with a smaller number of marked occurrences. The author provides a rumour dataset from Sina Weibo COVID-19 and offers a short, multimodal fusion model to detect rumours. A considerable improvement in rumour identification was observed on the Weibo and public PHEME datasets acquired by the model [25].

To achieve relatively close news accuracy of a classification and decrease root-mean-square error, the researchers used an actual GitHub dataset framed by COVID-19 news-related parameters. The deep learning classification schema developed the system with the greatest f1 score, which delivers 90% information categorization effectiveness [26, 27]. The author discusses methods of making existing and future approaches to the NLP more inclusive, including alternate methodologies, using off-the-box technologies, and establishing meaningful collaborations. The author proposes some guidelines for researchers who want to maximize the beneficial social effects of NLP [13, 28].

Authors work on four fundamental tasks of the NLP: retrieval of information, identification of named entities, literature-based discovery, and answering questions. The author also discusses four additional tasks directly addressing elements of the pandemic: topical modelling, sentimental and emotional analysis, predictive caseloads, and identification of disinformation. Finally, the authors highlight observed trends and difficulties [29].

The epidemic spreads, and more individuals seek COVID-19 testing and therapy. This cybercrime problem will probably persist. Information intelligence can improve the removal and prevention of harmful material to public authorities, regulators, legitimate manufacturers, and technological platforms [30].

The author presents a CORD19STS dataset to resolve this gain, which contains 13,710 sentence pairings that have been taken from the COVID-19 Open Research Dataset (CORD-19) challenge. In particular, the author produced a thousand pairs of sentences using various sample methods. The author utilized a fine-tuned BERT-like language model called Sen SCI-CORD19-BERT to compute similitude values between phrase pairings, which offers us an overall total of 32K phrase pairs to provide a balanced dataset for various semantical similar levels [31].

The evaluation encompasses about 150 NLP research and around 50 COVID-19 datasets and systems. Author's work on four fundamental tasks of the NLP: retrieval of information, identification of named entities, literature-based discovery, and answering questions. The author also discusses four additional tasks directly addressing elements of the pandemic: topical modelling, sentimental and emotional analysis, predictive caseloads, and identification of misinformation [29].

The research [32] reviews many documents that address similar problems with false news, sentiments categorization, and topics extraction. In the article, researchers are directed to valued practices to assist public authorities to fight the increase in falsification and harmful and hatred remarks, which may help enhance present research on COVID-19-related datasets [32].

Like other algorithms in natural language processing, it was suggested that the media articles be categorized as a dataset to evaluate the effects of COVID-19 pandemics in various sectors of the world economy. The model's accuracy was investigated based on the consistency and perplexity score, using LDA algorithms 0.51 and −10.90. Both the algorithm LDA and NMF found common issues in many areas of industry that were affected by the COVID-19 epidemic [33].

This study aims to create a natural language processing pipeline that can recognize patient information based on guidelines, and annotate it with Unify Medical Language Systems ideas for manual physician evaluation. The Human Abstraction, 2513 German clinical notes from the electro-heath report, is the gold standard for one-time evaluation. Clinical decision assistance systems might be developed by identifying recommendations from narrative clinical notes [34].

In this research, the author presents an automated summary assessment model (ASE), which is strictly dependent on the characteristics of the source text or the synthesis, which makes a quality model entirely text based. Summaries with accuracy above 80% are successfully classified as low or high quality. The model was created especially on many source texts, which allows for generalization across the text [35].

In 1964, PubMed and EMBASE data search was restricted to 27 suitable items. Data were collected for each research, purpose, the corpus of free texts, patients, symptoms, NLP technique, measurement metrics, and quality indicators. Future NLP research in EHR free-text narratives should study symptoms and symptom documentation. Investigating patient features and publicly developing NLP or pipelines and vocabulary algorithms linked to symptoms [36].

Patients who had chest CTs from 2000 through 2016 were found by interrogating institutional databases at a major quaternary referral centre. Using NLP, imaging reports were identified using GGOs, and further population data were obtained. The NLP examined a broad sample of individuals who had CT chests throughout the research. Provision for age, sex, race, and profession is the demographic characteristic of the GGOs reported [37].

In the Coronaviridae family, COVID-19 (Coron Virus Diasease-2019) is a member. No known treatment for an infectious disease wreaks havoc in people's lives and economic and financial institutions throughout the globe. Svc, KNN + NCA, Decision Tree Classifiers, and Naïve Bayes Bayesian Classifiers were all surpassed by Random Forests Regressor and Classifier [38].

According to one research, calcium channel blockers were linked to lower in-hospital mortality in patients with COVID-19 infection. The particular discovery was made possible by rapidly tailoring an NLP pipeline to the illness domain. Treatment effects previously undetectable by statistical means were discovered when that information was combined with already structured data [39].

Governments can make better choices if they can correctly predict the number of people infected with this virus. Few hybrid forecasting methods are proposed in this research for the COVID-19 time series. Each model may have different parameters, and Bayesian optimization makes it easier to predict future outcomes. Experiment findings show that deep learning models outperform the benchmark model in short- and long-term predicting scenarios [40, 41].

It would be helpful to have a technology that can correctly identify key COVID-19 clinical ideas from the free language in electronic health data to speed up clinical research. The COVID-19 SignSym was rapidly built using a hybrid method that combined deep-learning-based models with selected lexicons and pattern-based rules. Sixteen healthcare institutions currently use the technology publicly available to researchers as a downloadable package (https://clamp.uth.edu/covid/nlp.php) [42].

For finding positively diagnosed COVID-19 patients, VA built a Natural Language Processing pipeline and implemented it to speed up chart reviews. The system's accuracy is assessed at 82.4%, while its recall is calculated at 94.2%. Open-source code for a public-facing implementation has been made accessible to the public. So far, this approach has identified 36.1% of all confirmed positive cases in VA as part of the VA national response [43, 44].

In the last several months, the COVID-19 pandemic lockdown and hashtags were all over social media, with good and negative emotions expressed. Denmark and Sweden, for example, had opposing views on the government decision. However, unlike their South Asian neighbours, where people exhibited fear and anger, their nation's support was almost universal. The author found a new and innovative method to validate Twitter tweets extracted and analysed using supervised deep learning models [45].

Artificial intelligence has proven its capabilities in Natural Language Processing, Computer Vision, Bio-Informatics, Genetics, and Medical Sciences. The main reasons for such achievements are the availability of high computational resources and the rise of the big data era. However, it requires a deeper comprehension of relevant literature and resources to develop AI systems that help humanity in the real world. Experts in AI have a cross-domain knowledge to solve even simple tasks. Likewise, non-AI professionals have to dig deeper into AI literature to leverage AI capabilities for their domains. Time is a precious resource whose limitation can turn small obstacles into a big disaster, as observed in the COVID-19 epidemic. We believe that widespread infectious cases would be controlled if AI-driven medical-assistive tools could be developed in their early phase. As per our findings, no appropriate open-source platform offers AI services for a medical researcher to come up with exploration, prediction, or classification outcomes to overcome underlying challenges.

## 3. Methodology and Data Collection

### 3.1. Method

The data are taken from the study [24] and https://github.com/MickeysClubhouse/COVID-19-rumor-dataset. The summary of the process is explained below for the readers' information and knowledge. The rumours data are gathered from several sources such as Twitter discussions, which featured real-time arguments with specific tags, for data collection. To collect information from Tweets, the author concentrates on a few important hashtags and official accounts (such as NBC, Reuters, CNN, and News Channel) to track the latest developments on hot issues. The author includes the rumour phrases in the database and enhances the dataset with other information, like the website source, the publication date, the validity, the emotion, and the position taken in response to the rumour. The author also records any posts and comments or tweeting of the rumours and the stances taken by the people who shared them. The author divides the gathered rumours into two datasets depending on their source: (i) A bulletin dataset that contains rumours gathered from bulletin sites, and (ii) a tweet dataset that contains rumours gathered from social media platforms such as Twitter.

#### 3.1.1. Data Collection

**Figure 3:**
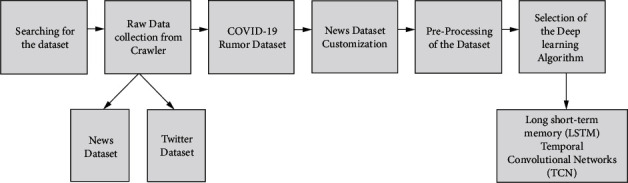
Flowchart depicting the collecting, labelling, and postprocessing of datasets.

Cheng et al. [24] have created website crawlers that consistently harvest information from the Chrome browser and Twitter.  Figure 3 displays the data collection method and the timeline for completion.

#### 3.1.2. Tweets Gathering Model

Using COV-19-related hashtags, including COV-19 and coronavirus, Cheng et al. gathered and recorded tweets on COVID-19 in the database. CSV files are available inside fake ID formats, issue date, and full text. Duplicate tweets were removed from the system. The emotion associated with each rumour is then determined via thoroughly examining the feeling elicited by the rumour's context. Furthermore, the author got the information associated with each tweet, including the text of the reply/retweet remark, the retweet number, the reply number, the like number, and the date the tweet was published. The metadata are then stored in separate files named after the fake IDs used in the tweets.

#### 3.1.3. News Collecting Method

Cheng et al. gathered comprehensive information on chosen news items from the Google browser; the authors utilize the mitmproxy method that enables HTTPS proxy. It is an interactive HTTPS proxy that is free to use. In contrast to the Tweet crawler, the search engine crawler primarily collects information from the page of search results. It searches the relative and absolute paths of the results based on the URL and timestamp. The author saved the rumours about breaking news in the *news.csv* file; in the *rumourID.csv* file, the author stored the rumours about reposts. Each rumour record includes a truthfulness label and the rumour's substance, and each reposting record includes a repost date, a repost website, and a stance tag, among other things. *It is important to note that not every source online has up-to-date material on the subject*.

#### 3.1.4. News Dataset Customization

Cheng et al. [24] combined the news dataset with the COVID-19 rumour dataset. “Very Negative (0), Negative (1), Neutral (2), Positive (3), and Very Positive (4)” are among the five label classes in the dataset. This dataset contains hundreds of news items for various labels. So, based on the classifications, the author scraped a new dataset from Google and combined it with the COVID-19 rumour dataset. The author has now employed five classes in the news dataset, as well as statistical data from the dataset, as shown below:

- “0–1159”
- “1–1895”
- “2–1079”
- “3–3536”
- “4–1531”

#### 3.1.5. Data Records

**Figure 4:**
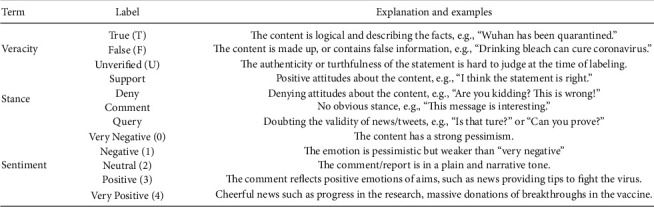
Labels in the dataset are highlighted—adopted from [24].

All data are stored in.csv files. For the greatest display, it is recommended that we utilize Utf-8 encoding. Two datasets are created to contain rumours gathered from news and Twitter.  Figure 4 lists the tag definitions and provides a collection of information for future reference and research.

### 3.2. Datasets

#### 3.2.1. News Dataset

Information from news stories on COVID-19 is included in the news dataset. It includes information on emergencies, public figures' remarks, updates on the coronavirus epidemic, and other information. Each record includes the following structured metadata, which describes the specifics of the news story:

- *Sources*: Some websites include the rumour phrase. The rumour's origin is determined by counting the number of websites that discuss it, such as those that debate its validity. The oldest rumour source is indicated as the source of the rumour.
- *Popularity*: The number of websites that repost the entire rumour in the Chrome browser shows the rumour's popularity.
- *Date*: The date on which each rumour record was published, as determined by the web crawler, is mechanically gathered.
- *Stance*: The mindset of the rumour source's author or editor is important to note. Our categorization system follows the traditional classification system and divides rumour attitude into four categories: support, denial, remark, and inquiry. The positions are labelled and cross-validated manually, after which they are checked against the context of each page. It is worth mentioning that the majority of the positions fall into the support and comment categories.
- *Sentiment*: To identify a rumour term, we need one of five distinct sentiments: very (negative), negative, (neutral), (positive), or (very positive). The author Cheng et al. carefully classify and cross-validate the feeling to decide whether this is bad or good news, depending on the circumstances. News articles that report: (i) COVID-19 cases are categorized as Negative news; (ii) COVID-19-related deaths are categorized as very negative news; (iii) COVID-19-related prevention tips are categorized as positive news; (iv) COVID-19-related campaigns and vaccine advancement are categorized as highly positive news.
- *Veracity*: True or false, indicating that the data are describing a reality; unverified, indicating that the news has not been verified as of the time of collection; or true or false, indicating that the news has not been confirmed as of the time of collection. The labelling and cross-validation are done manually during the data collection stage, using reputable sources and widely shared common knowledge [24].

#### 3.2.2. Twitter Data

The Twitter dataset includes speculations that have been published on the social media platform. The data are compiled from public accounts which have commented on COVID-19-related information discussion forums that have been labelled with COVID-19-related tags. Other users on the social media site may retweet or reference the discussions.

- In the same way, as sources in a news dataset shows how individuals react to a tweets (from social media twitter), responses indicate what people react to the tweet.
- Reply/Retweet/Like (RRL) number: These figures show the trend in the spread of a tweet. The crawler automatically parses the RRL number and displays it.
- Popularity: When you add up the RRL numbers, you get an idea of how popular a tweet is.
- Data: In the Twitter platform, the date is expressed using MM.DD.YYYY, representing when the tweet was published on the platform [24].

### 3.3. Preprocessing Steps

1. Read the dataset
2. Removal of stop words
3. Removal of symbols
4. Removal of digits
5. Tokenization from each row
6. Making a vector shape
7. Pass vector from module

## 4. Results and Discussion

### 4.1. Experiment 1

#### 4.1.1. Long Short-Term Memory (LSTM)

**Figure 5:**
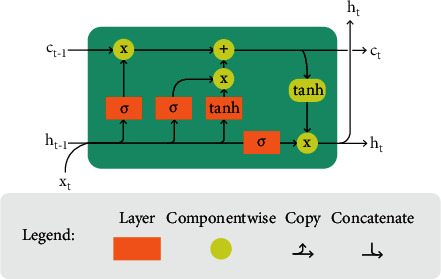
Schematic of the long short-term memory cell—adopted from [47].

LSTM is an in-depth learning artificial recurrent neural network architecture (RNN). In contrast to conventional neural networks, feedback connections are available to LSTM. It can handle single data points, like pictures, and whole data sequences, like voice or video [46]. For example, LSTM is for tasks like unsegmented handwriting recognition, voice recognition, and network traffic or IDS anomaly detection, as shown in Figure 5.

#### 4.1.2. Temporal Convolutional Networks (TCN)

**Figure 6:**
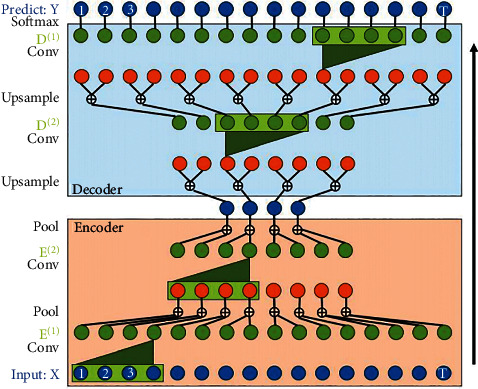
Schematic of the Temporal Convolutional Networks—adopted from [50].

The first major study suggested a TCN for the segmentation of video-based actions. The two stages of this classic approach include calculating low-level CNN characteristics that encode space-time information and introducing those low-level characteristics into a ranking that collects time information on a high-level basis using RNN [20, 48, 49]. A similar method needs two distinct models, which is the major drawback. TCN offers a unified method to hierarchically capture all two information layers (encoder-decoder), as shown in Figure 6.

We discussed the analysed findings from several deep learning algorithms in the results section, which we applied to the “news from Google and Twitter” dataset. We employed Keras innovative models “Long short-term memory (LSTM) and Temporal Convolutional Networks (TCN)” in this study. We gathered 9200 Google comments and 34,779 Twitter postings filtered for phrases connected with bogus news about the COVID-19. The dataset was assessed using three criteria: truthfulness, stance, and sentiment. The analysis results reveal which model has the best accuracy and loss rate. Precision, f1 score, and recall are the additional characteristics. We utilized it to verify the deep learning model's legitimacy.

#### 4.1.3. The Evaluated Results for Sentiment and Veracity

We analysed the dataset of Google rumour news on the COVID-19 in this area. This dataset was used in two ways: sentimentality analysis and truthfulness analysis. “Very Negative (0), Negative (1), Neutral (2), Positive (3), and Very Positive (4)” are the sentiment labels we utilized. “True (T), False (F), and Unverified (U)” is also the veracity. For the analysis, we used 9200 rumour news after preprocessing against COVID-19. A total of two methods for deep learning were used in this study: “Long short-term memory (LSTM)” and “Temporal Convolutional Networks (TCN).” We compared them and determined which one is the best for real-time implementation.

*(1) Compiling Model*. The initial stage is to give the input dataset to several models. The computational graph represents the dropout and layers involved in the model. These models have been trained and tested, and their performance has been quantified in terms of loss and accuracy. The compared models are implemented using the following hyperparameter settings.

- Batch Size: 5
- Epoch: 10
- Optimizer: “Adam”

**Figure 7:**
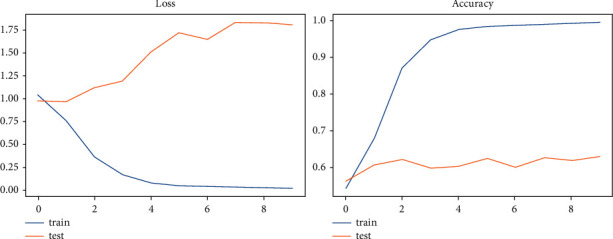
LSTM—the graphical representation of evaluating results for sentiment dataset.

**Figure 8:**
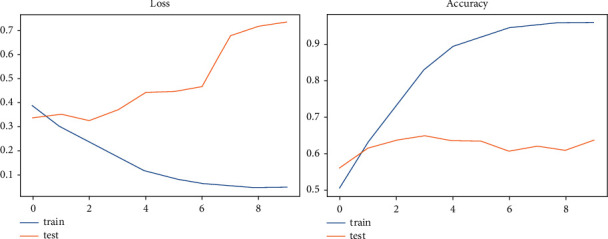
TCN—the graphical representation of evaluating results for sentiment dataset.

Each of the models examined in this study is represented in Table 1 by its accuracy and loss rate, with the TCN model having the best val_loss and val_accuracy of 0.7345 and 63.59%, respectively. We showed that the TCN model had the best evaluated results for the sentiment dataset since other models' accuracy and loss rate were included in the table. For each sentiment model, accuracy and loss are shown graphically in Figures 7 and 8.

**Figure 9:**
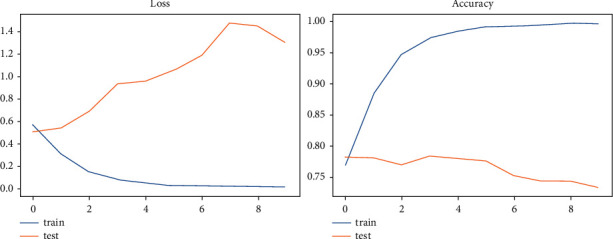
LSTM—the graphical representation of evaluating results for veracity dataset.

**Figure 10:**
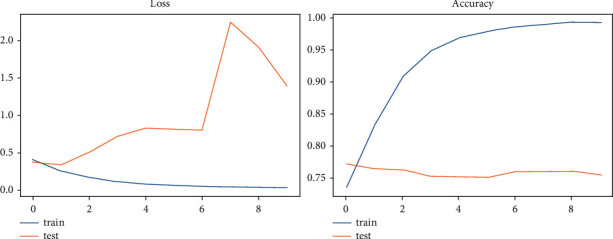
TCN—the graphical representation of evaluating results for veracity dataset.

Table 2 shows the accuracy and loss rate of each model we employed in the investigation, with the TCN model having the best val_loss and val_accuracy of 1.3806 and 75.43%, respectively. According to the veracity dataset's accuracy and loss rate of other models, we found that the TCN model had the best evaluated results. Figures 9 and 10 depict a graphical depiction of the accuracy and loss of all of the veracity models we have developed.

Consequently, our dataset is unbalanced, with 346 of the 920 instances falling into one of the three categories. For each approach, the ratio is 0.73%. Since the predictor is mostly right when it comes to class 3 samples, it has an incredibly high level of precision, recall, and f1-score values for class 3 and extremely low scores for the other classes. Macro *F*1's objective is to calculate the *F*1 split by class without using weights for the aggregate:

(1) $$\begin{array}{c} F_{\text{class}1}+F_{\text{class}2}+F_{\text{class}3}+F_{\text{class}4}. \end{array}$$

You will be penalized if the model fails to perform effectively among minority groups (which you want when there is an imbalance).

It is calculated by the number of true labels in each class when *F*1 costs are merged:

(2) $$\begin{array}{c} F_{\text{class}1}*C1+F_{\text{class}2}*C2+F_{\text{class}3}*C3+F_{\text{class}4}*C4. \end{array}$$

Consequently, you prefer the class labels (which you typically would not want).

Therefore, our modelling is incorrect for one of the classes since the macro F1 score correctly captures but is not weighted, leading to the five gaps in our dataset, as shown in Table 3. The TCN model has high precision, recall, F1 score from other models, and accuracy of 64%.

As a result, our dataset is skewed, with 456 out of 920 instances falling into the *T* group (0.81% for various techniques, respectively). These results in exceptionally high precision, recall, and f1-score values for class *T*, and extremely low scores for the other classes, as a consequence of the predictor virtually always accurately predicting any given sample from class *T*. Still, weighted does not result in a mismatch between the three classes in your model. Based on these data, Table 4 shows that the TCN model has a high accuracy of 75% compared to other models.

#### 4.1.4. The Evaluated Results for Stance

We analysed the dataset of Google rumour news on the COVID-19 in this area. This dataset was used in two ways: sentiment and truthfulness. “Deny, Comment, Query, and Support” are the sentiment labels we have utilized. We used the 34,779 twitter comments against the COVID-19 after preprocessing for the analysis. For the investigation, we employed two deep learning algorithms: “Long short-term memory (LSTM)” and “Temporal Convolutional Networks (TCN).” We compared them and determined which one is the best for real-time implementation.

**Figure 11:**
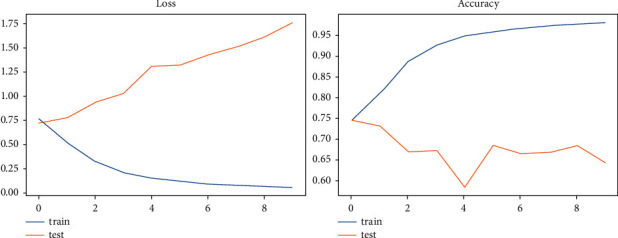
LSTM—the graphical representation of evaluating results for stance dataset.

**Figure 12:**
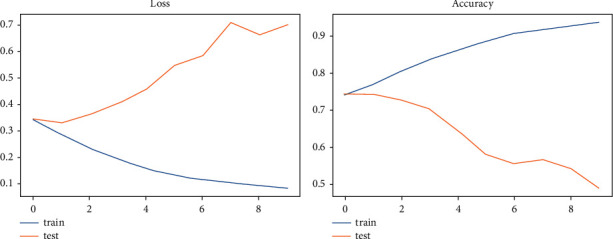
TCN—the graphical representation of evaluating results for stance dataset.

The accuracy and loss rate of each model we utilized for the analysis are shown in Table 5. We found that the LSTM model has 64.20% greater accuracy than the TCN model but has more val_loss, consistent with epoch 4. As a result, we determined that the TCN model had the best val_loss and val accuracy, with values of 0.6985 and 48.96%, respectively. We examined that the TCN model has the best-evaluated results for the stance dataset since other models' accuracy and loss rates are shown in Table 5. Figures 11 and 12 depict a graphical depiction of the accuracy and loss of all the deployed stance models.

Consequently, our dataset is unbalanced, with the Deny class accounting for 2462 of the 3478 cases (0.77 and 0.64% for various methods, respectively). Because of this, the class Deny has extraordinarily high accuracy, recall, and f1 scores, while the other classes have extremely poor accuracy, recall, and f1 scores. You can see this in your macro F1 score (correct), but weighted (inaccurate) shows the four-class disparity.  Table 6 demonstrates that the TCN model has good accuracy, recall, and F1 score compared to other models and a 49% success rate.

### 4.2. Experiment 2

Table 7 illustrates the sentiment dataset which has been divided into five classes and each class is assigned a specific numerical label value ranging from 0 to 4 describing the extent of how positive or negative the news is. All the classes show varying dataset lengths among which class 2 depicts the lowest length (1079) while class 4 depicts the highest length (1531). The description of all the labels is as follows:

- 0 = very negative
- 1 = negative
- 2 = neutral
- 3 = positive
- 4 = very positive

Table 8 illustrates the stance dataset which is divided into four classes under the labels of comment, support, query and denies. The labels showed significant variations in lengths. The Comment label showed the highest length (24222) while the Deny label exhibited the lowest length (1750).

Table 9 demonstrates the veracity dataset which is divided into four classes including true (*T*), false (*F*), unverified (*U*), and Twitter (*U*). The highest length was shown by *T* class (4485) while the lowest length was depicted by Twitter (*U*) (1).

The datasets presented above showed multiclass division. Therefore, the current study also employed multiclassification models for the evaluation of the datasets. The current study employed several state-of-the-art deep learning models for this purpose.

#### 4.2.1. Training Architecture of Deep Learning Models

The study used the following deep learning models:

- Simple RNN Architecture
- LSTM + Word Embedding (WE)
- Bidirectional + Word Embedding (WE)
- LSTM + CNN-1D

**Figure 13:**
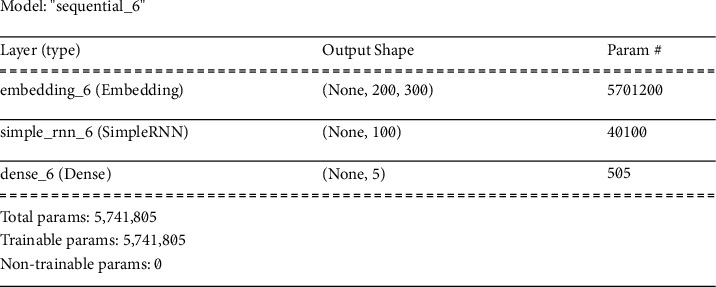
Summary of simple RNN model.

The architecture of the important deep learning models is presented below:

*(1) Simple RNN Architecture*. It is a form of neural network in which the nodes are connected with each other. This connection exists along a temporal sequence which refers to the transition of data along with time. This neural network is preferred over conventional neural networks. The reason lies in the capability of this model to process the past input to address the future inputs. For instance, RNN models have the capacity to predict the next word by analysing the previous words present in the sequence while a conventional model lacks this capability. The RNN models recur the information in a loop which makes it possible for the information to remain in the system.  Figure 13 demonstrates the detailed summary of the simple recurrent neural network (RNN) model architecture. The first layer is the embedding layer for which the calculated parameters are 5701200. The next layer is the simple RNN layer for which we calculated 40100 trainable parameters. The additional 505 parameters added in the total param count were due to the dense layer added after the simple RNN layer.

**Figure 14:**
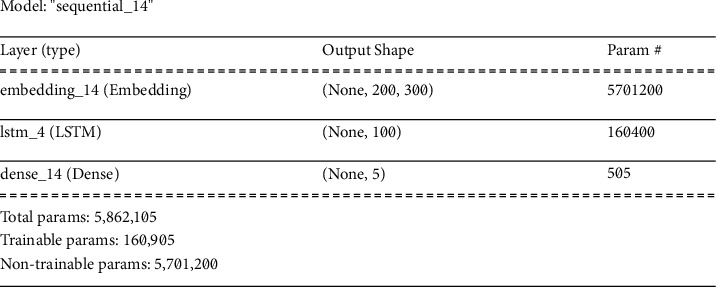
Summary of LSTM + Word Embedding model.

*(2) LSTM* *+* *Word Embeddings Architecture*. Long short-term memory (LSTM), a type of recurrent model, is a widely used model in deep learning. This model is highly advantageous because it utilizes both the previous and future memory of the available data. Moreover, its performance in the case of time-series data is also quite effective. We have used Global Vectors for Word Representation (GloVe) for word embeddings which is an unsupervised learning algorithm. These are Google-made word embeddings with almost 800 billion words and 300-dimensional embeddings of them (see Figure 3).  Figure 14 shows the detailed model summary of the LSTM + Word Embeddings model used on the dataset of this study. It has three layers including embedding, LSTM, and dense layers. The embedding layer has the same number of parameters as in the previous model (5701200) but they are treated as nontrainable parameters in this model while the dense layer has 505 trainable parameters. However, this model has a different count of the total number of parameters than the previous model because the LSTM layer has 160400 parameters that increase the total number of trainable parameters to 5862105.

**Figure 15:**
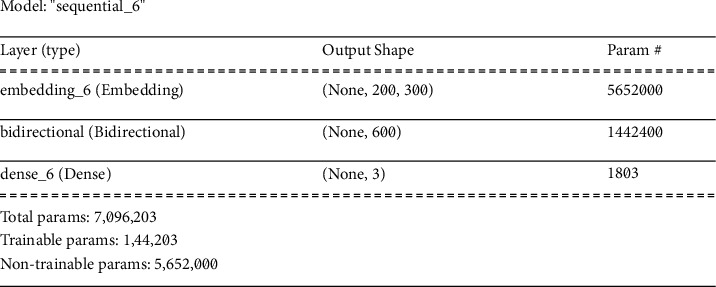
Summary of Bidirectional + Word Embedding model.

*(3) Bidirectional* *+* *Word Embeddings Architecture*. We employed the bidirectional feature in combination with word embeddings to function on both the right and left sides of the text dataset. This kind of model works efficiently in cases where the text dataset is quite large and the purpose is to create a summary of the dataset.  Figure 15 illustrates the summary of the Bidirectional + Word Embedding model. The model entails three layers, i.e., embedding, bidirectional, and dense layers. The first layer is the embedding layer that has 5652000 nontrainable parameters. The next layer is the bidirectional layer that has 1442400 trainable parameters. The third layer (dense layer) provides additional 1803 parameters, taking the total count of parameters to 7096203.

**Figure 16:**
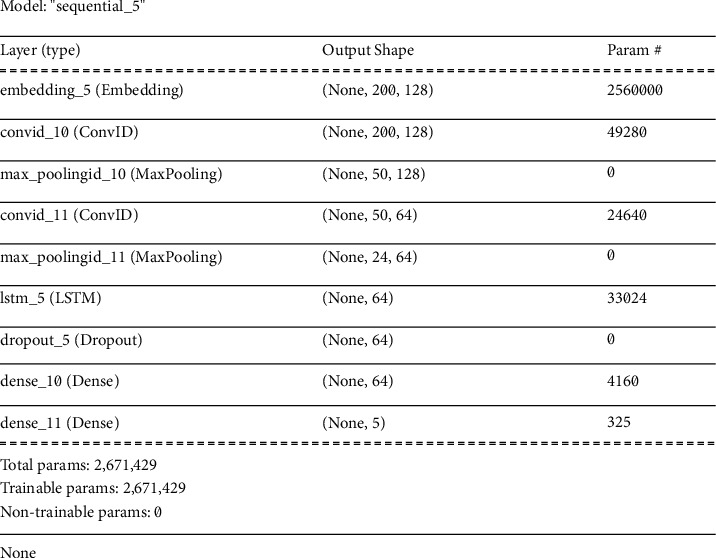
Summary of LSTM + CNN-1D model.

*(4) LSTM* *+* *CNN-1D Architecture*. It is important to understand the use of both CNN and LSTM to comprehend their combined model use in this study. CNNs have been extensively utilized in modelling issues in relation to inputs, such as image datasets. Over the years, CNNs have provided a great opportunity to detect and classify the image dataset to extract vital information from the dataset. On the other hand, LSTMs are employed in tasks where the dataset has a sequence and they perform predictions based on the sequence. It assists in undertaking those tasks that require image sequences to predict certain information for which a more sophisticated model is required. To this end, the LSTM + CNN model is utilized to predict spatial input such as images and videos. In this model, the task of feature extraction is undertaken using CNN while LSTM helps in prediction. Since the current model is applied to the text dataset, simply a one-dimensional (1D) model is employed.  Figure 16 demonstrates the model summary of LSTM + CNN 1D architecture. It contains six types of layers including embedding, Conv1D, max pooling, LSTM, dropout, and dense layers. The model has no nontrainable parameters. The first layer is the embedding layer with the highest number of trainable parameters (2560000) followed by the second layer Conv1D. The LSTM layer contains only 33024 trainable parameters while the max pooling has no trainable parameters that impact backpropagation.

### 4.3. Accuracy Performances of Deep Learning Models

We compared the classification accuracy performance of deep learning models with the BERT model. The accuracies of all deep learning models were high for all three datasets. Especially, for veracity datasets, all models showed >99% accuracy except BERT which showed 97.11% accuracy for veracity datasets. LSTM + CNN model showed the highest accuracy for the sentiment dataset (99.88%) while LSTM + word embeddings showed the lowest accuracy for the sentiment dataset (84.97%). For the stance dataset, LSTM + CNN showed the highest accuracy (99.96%) while BERT showed the lowest accuracy (92.57%). A detailed summary of the accuracy performance of all deep learning models for all three datasets is provided in Table 10.

### 4.4. Limitations and Validity Threats

#### 4.4.1. Limitations

One of the difficulties in building such a machine learning technique is validating many COVID-19 claims. The COVID-19 Fake News Detecting dataset, which is rather tiny, is the foundation for our approach. As a result, it may be restricted in its ability to identify new COVID-19-related disinformation. More evaluations are needed to improve the external validity of the used models. We will dig deeper into the data that we have gathered in the future and focus on higher-order metadata collecting.

#### 4.4.2. Validity Threats

Every study faces a threat to validity (https://1library.net/article/construct-validity-threats-validity-learning-structural-historical-features.z193jpvq). Below mentioned are some of the threats to validity.

- *Construct Validity* [51]: threats to construct validity centre on how theory and observation are related. This may be interpreted in our context as referring to the validity of the models that were trained and used to assess the various methodologies examined in this work. By reviewing and reevaluating with other coauthors, we have attempted to reduce any threat to construct validity. To further validate and draw conclusions from the data, more assessments are required.
- *Internal Validity* [52]: All the variables that may have influenced our results are threats to internal validity. Even though we compared the suggested process with traditional deep learning techniques when conducting experiments, in our context, this may apply to the training procedure described in Section 5. More comparisons, assessments, and reevaluations are required to validate the study's findings further.
- *External Validity* [51, 53]: threats to external validity concern the generalizability of the findings. Further extensive evaluations on selected and new DL models are required to reduce this threat.

## 5. Conclusion and Future Work

Since December 2019, the (COVID-19) coronavirus outbreak has aroused enormous worry within the general community and dramatically changed social attitudes and beliefs. Besides the sickness, many people suffer from anxiety and sadness from the disease. Rumours are unconfirmed facts or stories that disseminate disinformation and generate attitudes of prejudice, hatred, and fear. In this research study, we took a COVID-19 rumours dataset from news websites and tweets, combined with metadata regarding the rumours. We have collected 9200 comments from Google and 34,779 Twitter postings filtered for terms linked with COVID-19-related fake news. In experiment 1, the dataset was examined using the following three criteria: truthfulness, posture, and sentiment. In these words, we have distinct labels and performed these deep learning algorithms independently on each term. The TCN model performs best on each measurement parameter in the examined findings. So, we have adopted the TCN model for the practical implication of improved results. In experiment 2, we employed multiple state-of-the-art deep learning algorithms and examined the performance of these models on all three datasets. Based on our experimental assessments, the BERT performs better than the other state-of-the-art models assessed in our study. Some of the future directions for the fake news topic are as follows:

- Rumour processing and emotional research in NLP
- Conspiracy processing and emotional study in NLP
- Recognition, prediction, rumour, and biased news categorization
- Social media network and challenging information flow study, as well as transitional network-related research

## Figures and Tables

**Table 1:** The evaluating results of the sentiment dataset.

Long short-term memory (LSTM)			Temporal Convolutional Networks (TCN)		
Epoch	Val_Loss	Val_Accuracy	Epoch	Val_Loss	Val_Accuracy
1	0.9743	0.5598	1	0.3340	0.5598
2	0.9648	0.6043	2	0.3490	0.6152
3	1.1173	0.6196	3	0.3237	0.6359
4	1.1919	0.5967	4	0.3680	0.6478
5	1.5083	0.6022	5	0.4400	0.6348
6	1.7218	0.6239	6	0.4447	0.6337
7	1.6471	0.6000	7	0.4657	0.6065
8	1.8342	0.6250	8	0.6769	0.6196
9	1.8323	0.6174	9	0.7163	0.6087
10	1.8093	0.6293	10	0.7345	0.6359

**Table 2:** The evaluating results of the veracity dataset.

Long short-term memory (LSTM)			Temporal Convolutional Networks (TCN)		
Epoch	Val_Loss	Val_Accuracy	Epoch	Val_Loss	Val_Accuracy
1	0.5035	0.7815	1	0.3569	0.7717
2	0.5381	0.7804	2	0.3208	0.7641
3	0.6837	0.7696	3	0.4927	0.7620
4	0.9285	0.7837	4	0.7023	0.7522
5	0.9540	0.7793	5	0.8169	0.7511
6	1.0478	0.7750	6	0.7995	0.7511
7	1.1807	0.7522	7	0.7916	0.7598
8	1.4689	0.7435	8	2.2497	0.7587
9	1.4431	0.7435	9	1.9047	0.7598
10	1.2965	0.7326	10	1.3806	0.7543

**Table 3:** The average evaluating results of sentiment dataset.

<!—Col Count:9	Long short-term memory (LSTM)				Temporal Convolutional Networks (TCN)			
	Precision	Recall	F1 score	Support	Precision	Recall	F1 score	Support
0	0.52	0.39	0.45	112	0.56	0.48	0.52	112
1	0.65	0.69	0.67	197	0.62	0.70	0.66	197
2	0.47	0.46	0.47	117	0.44	0.47	0.45	117
3	0.69	0.78	0.73	346	0.71	0.75	0.73	346
4	0.62	0.52	0.56	153	0.71	0.54	0.61	153
Accuracy			0.63	920			0.64	920
Macro avg	0.59	0.57	0.58	920	0.61	0.59	0.59	920
Weighted avg	0.62	0.63	0.62	920	0.64	0.64	0.63	920

**Table 4:** The average evaluating results of the veracity dataset.

	Long short-term memory (LSTM)				Temporal Convolutional Networks (TCN)			
	Precision	Recall	F1 score	Support	Precision	Recall	F1 score	Support
*F*	0.85	0.74	0.79	343	0.80	0.84	0.82	343
*T*	0.79	0.82	0.81	456	0.85	0.77	0.81	456
*U*	0.32	0.40	0.36	121	0.37	0.45	0.41	121
Accuracy			0.73	920			0.75	920
Macro avg	0.65	0.65	0.65	920	0.67	0.69	0.68	920
Weighted avg	0.75	0.73	0.74	920	0.77	0.75	0.76	920

**Table 5:** The evaluating results of the stance dataset.

Long short-term memory (LSTM)			Temporal Convolutional Networks (TCN)		
Epoch	Val_Loss	Val_Accuracy	Epoch	Val_Loss	Val_Accuracy
1	0.7194	0.7432	1	0.3461	0.7430
2	0.7765	0.7303	2	0.3302	0.7418
3	0.9348	0.6691	3	0.3602	0.7266
4	1.0246	0.6722	4	0.4021	0.7033
5	1.3052	0.5828	5	0.4575	0.6455
6	1.3175	0.5828	6	0.5456	0.5808
7	1.4237	0.6650	7	0.5838	0.5555
8	1.5037	0.6676	8	0.7077	0.5664
9	1.6065	0.6834	9	0.6613	0.5428
10	1.7549	0.6420	10	0.6985	0.4896

**Table 6:** The average evaluating results of stance dataset.

	Long short-term memory (LSTM)				Temporal Convolutional Networks (TCN)			
	Precision	Recall	F1 score	Support	Precision	Recall	F1 score	Support
Deny	0.78	0.76	0.77	2462	0.78	0.54	0.64	2462
Comment	0.32	0.30	0.31	193	0.41	0.09	0.15	193
Query	0.19	0.19	0.19	261	0.08	0.40	0.14	261
Support	0.39	0.47	0.43	562	0.53	0.43	0.48	562
Accuracy			0.64	3478			0.49	3478
Macro avg	0.42	0.43	0.42	3478	0.65	0.65	0.35	3478
Weighted avg	0.65	0.64	0.65	3478	0.75	0.73	0.55	3478

**Table 7:** Statistics of sentiment in the COVID-19 dataset.

Classes	Length
0	1158
1	1895
2	1079
3	3536
4	1531

**Table 8:** Statistics of stance in the COVID-19 dataset.

Classes	Length
Comment	24222
Support	5248
Query	2474
Deny	1750

**Table 9:** Statistics of veracity in the COVID-19 dataset.

Classes	Length
*F*	3460
*T*	4485
*U*	1253
*U* (twitter)	1

**Table 10:** Results of accuracy performance of deep learning models and BERT for sentiment, stance, and veracity datasets.

Models	Dataset	Accuracy (%)	Val accuracy (%)	Loss	Val loss
Simple RNN	Sentiment	97.80	60.49	0.0575	0.4175
	Stance	99.12	76.03	0.0790	0.2790
	Veracity	99.97	66.66	0.0309	0.3009
LSTM + WE	Sentiment	84.97	83.90	0.0871	0.0871
	Stance	96.10	89.13	0.02789	0.02789
	Veracity	99.03	94.02	0.0211	0.0211
Bidirectional + WE	Sentiment	92.5	93.08	0.0108	0.0108
	Stance	97.60	83.89	0.1709	0.1709
	Veracity	99.97	98.93	0.00928	0.00928
LSTM + CNN	Sentiment	99.88	63.08	0.0108	0.0108
	Stance	99.96	73.89	0.1709	0.1709
	Veracity	98.96	78.93	0.00928	0.00928
BERT	Sentiment	99.18	98.17	0.0308	0.0302
	Stance	92.57	96.89	0.1710	0.0959
	Veracity	97.11	99.01	0.1226	0.0210

## Data Availability

The datasets presented in this study can be found in the online repository https://github.com/MickeysClubhouse/COVID-19-rumor-dataset.
